# Supplementary material for: Independent transcriptional patterns reveal biological processes associated with disease-free survival in early colorectal cancer
Source: Commun Med (Lond). 2024 May 3;4:79. doi: 10.1038/s43856-024-00504-z (PMC11068726; doi:10.1038/s43856-024-00504-z)
Supplement: Supplementary file 2 — Supplementary Files [file 43856_2024_504_MOESM2_ESM.pdf]

Supplementary Information

Daan G. Knapen[1], Sara Hone Lopez[1], Derk Jan A. de Groot[1], Jacco-Juri de Haan[1], Elisabeth G.E. de Vries[1], Rodrigo Dienstmann[2], Steven de Jong[1], Arkajyoti Bhattacharya[1], Rudolf S.N. Fehrmann[1] Independent transcriptional patterns reveal biological processes associated with disease-free survival in early colorectal cancer

Supplementary Methods.

Supplementary References.

Supplementary Figure 1. Random survival forest analysis of stage 2 early colon cancer patients, optimal clusters.

Supplementary Figure 2. Random survival forest analysis of stage 3 early colon cancer patients, optimal clusters.

Supplementary Figure 3. Clustering results of Random survival forest analysis of stage 2 early colon cancer patients.

Supplementary Figure 4. Clustered activity of EMT-related DFS-associated TCs

Supplementary Figure 5. Spatial transcriptomic profiles in colorectal cancer samples

Supplementary Figure 6. The influence of mesenchymal cell types on EMT-related DFS-associated TCs

Supplementary Table 1. Pairs of TCs

Supplementary Table 2. Important scores TCs

This supplemental material has been provided by the authors to give readers additional information about their work.

Supplementary Methods.

Data Acquisition

Publicly available raw microarray expression profiles were extracted from the Gene Expression Omnibus (GEO).[1] The acquisition was restricted to the Affymetrix HG-U133 Plus 2.0 platform (GEO accession identifier: GPL570). Profiles were selected when generated with tissue samples of various healthy and disease conditions in the large intestine (Figure 1).

For all selected early colorectal cancer (CRC) samples, corresponding clinicopathological (CP) data were collected from GEO or the corresponding manuscript. CP data included disease-free survival (DFS) data, stage, primary tumor location, age, gender, microsatellite instability (MSI) status, received adjuvant treatment or not, tumor protein P53 (TP53) status, Kirsten rat sarcoma virus (KRAS) status, and v-Raf murine sarcoma viral oncogene homolog B (BRAF) status.

The pre-processed and normalized expression profiles were used to infer the composition of the tumor microenvironment using the Microenvironment Cell Population (MCP) counter v.1.1. R package.[2] MCP-counter infers the absolute abundance of eight immune and two stromal cell populations from bulk gene expression profiles. The MCP-counter computes scores per sample per cell type expressed in arbitrary units. These abundance scores allow the comparison of cellular composition between samples. The random forest CMS classifier was applied to the data set, and a CMS class was assigned when the posterior probability of a sample belonging to a subtype was ≥0.5.[3]

Sample processing and quality control

Non-corrupted raw data CEL files were downloaded from GEO for the selected samples. We generated an MD5 hash for each CEL file to identify samples uploaded to GEO multiple times. After removing duplicate CEL files, pre-processing and aggregation of CEL files was performed with robust multiarray averaging (RMA using the justRMA function from R package aroma.affymetrix v3.2.0) method using R version 3.5.2. Principal Component Analysis (PCA) on the sample correlation matrix was used for quality control as previously described.[4] The first principal component (PCqc) of such an expression microarray correlation matrix nearly always describes a constant pattern that dominates the data, explaining around 80% to 90% of the total variance. This pattern can be regarded as probe-specific or platform-specific variance, independent of the biological sample hybridized to the array. The correlation of each microarray expression profile with this PCqc can be used to detect outliers, as arrays of lesser quality will have a lower correlation with the PCqc. We removed samples that had a correlation $R < 0.8$. MD5 hash duplicate removal does not detect identical samples when meta-data is different in the raw CEL files. Pearson correlation coefficients among gene expression profiles were obtained to identify samples with identical expression values. One sample from each set of duplicate samples was randomly chosen, and the remaining were removed from subsequent analyses.

Consensus-independent component analysis (c-ICA)

Consensus-independent component analysis (c-ICA) was performed to segregate the average expression patterns of complex biopsies into statistically independent transcriptional patterns as previously described.[5] These patterns are hereafter referred to as transcriptional components (TCs).

Applying ICA on a gene expression data set with p genes and n samples results in the extraction of *i* independent components of dimension 1×*p* (hereafter called estimated sources, ESs) and a mixing matrix (MM) of dimension *i* × *n,* which contains the coefficients of ESs in each sample. The weight of each ES represents the direction and magnitude of its effect on the expression level of each gene. The coefficients of MM represent activity scores of the ESs in the corresponding sample. In ICA, a pre-processing technique called whitening is applied to the input data set to make the estimation more time-efficient. Whitening was used to transform the gene expression profiles of all samples so that the transformed profiles are uncorrelated and have a variance of one. Next, ICA was performed on the whitened data set using the fastICA algorithm in our in-house tool Analyzertool version 5.0, resulting in the extraction of *i* independent components and a mixing matrix. The parameter *i* was chosen as the number of top principal components, which captured 90% of the total variance in the data set.

In ICA, an initial random weight vector with a variance of 1 must be chosen to start the search for statistically independent ESs. Hence, varying initial random weight vectors could result in different sets of ESs. To retrieve a set of consensus ESs (referred to as TCs), we performed 25 ICA runs, each with another random initialization weight vector. The assumption is that over many ICA runs, the fastICA algorithm converges to global solution for most of the runs. ESs extracted from these runs were clustered together if the absolute value of Pearson correlation between them was > 0.9. Clusters with ES present in >50% of the runs were used to obtain *m* TCs using the following formula:

$$TC_{p\times 1} = (1/n)\sum_{i=1}^{n}\left(ES_i \times sign\big(correlation(ES_1, ES_i)\big)\right)$$

These *m* TCs and the gene expression data set with *p* genes and *n* samples ($X_{p\times n}$) were used in the formula below to obtain a consensus mixing matrix (or CMM), which contains the coefficients of TCs in each sample:

$$CMM_{m\times n} = ((TC')_{m\times p} \times TC_{p\times m})^{-1} \times (TC')_{m\times p} \times X_{p\times n}$$

The coefficients of CMM represent the activity scores of the TCs in the corresponding sample.

Survival analysis

Univariate survival analysis

In a subset of samples (n = 836, Table 1), we performed univariate and multivariate Cox proportional hazards analysis to assess the association between the activity of TCs and DFS. The activity of each TC was used as a predictor in each Cox proportional hazards analysis, which resulted in an adjusted p-value for the corresponding TC, thus conveying the significance of the association between the activity of the TC with DFS. Thereafter, -$\log_{10}$(adjusted p-value) for each TC i was obtained ($original\_minus\_log10\_adj\_p_i$) for further analyses. We conducted a permutation test to reduce the probability of false-positive association due to multiple testing. The permutation test was performed on each TC separately using the following steps:

1. The activity scores of the TCs are permuted 10,000 times.
2. Permuted activity of each individual TC is used as a predictor in each Cox proportional hazards analysis to find association with DFS. Each Cox proportional hazards analysis results into an adjusted p-value for the corresponding permuted TC.

3. From the cox proportional hazards analyses described above, $-\log_{10}$(adjusted p-value) corresponding to all permuted TCs are obtained (referred to as permuted_minus_$\log_{10}$_adj_p).
4. Sort the weights of permuted_minus_$\log_{10}$_adj_p in decreasing order (sp_minus_$\log_{10}$_adj_p).
5. Sort the weights of original_minus_$\log_{10}$_adj_p in decreasing order (so_minus_$\log_{10}$_adj_p).
6. For every weight of so_minus_$\log_{10}$_adj_p (so_minus_$\log_{10}$_adj_p_j), obtain the number of weights of sp_minus_$\log_{10}$_adj_p_i greater than so_minus_$\log_{10}$_adj_p_j ($f_{>j}$).
7. Obtain the optimal cut-off for the sp_minus_$\log_{10}$_adj_p_i (oc_i) as the maximum value of the weights of so_minus_$\log_{10}$_adj_p for which $f_{>j}/j > 1\%$.
8. Indicator marks (im) for every weight of the original_minus_$\log_{10}$_adj_p (original_minus_$\log_{10}$_adj_p_s) are obtained in the following way
   a. If original_minus_$\log_{10}$_adj_p_s >= 80% quantile of oc then $im_s = 1$
   b. Otherwise zero.
9. TCs with im as 1 were statistically significantly associated with DFS in the permutation test framework.

Multivariate survival analysis

Next, we performed a multivariate Cox proportional hazards analysis using gender, MSI status, BRAF status, KRAS status, primary tumor location, stage, and treatment with adjuvant systemic therapy as covariates. These were all categorical data. Therefore, if data was missing, another category was added with the label 'unknown' and these samples with

‘unknown’ labels were included in the analysis. For each TC, multivariate survival analysis with DFS as the outcome variable was conducted using the following steps:

1. Activity of each individual TC was used as a predictor in each Cox proportional hazards analysis, which resulted in an adjusted p-value for the corresponding TC. Thereafter, -$\log_{10}$ values (adjusted p-values) for each TC as a predictor (original_minus_$\log_{10}$_adj_p_i) were obtained from the multivariate survival analysis.
2. Permutation tests were performed on each TC, as described in the univariate survival analysis section. As a result, we obtained a list of TCs significantly associated with DFS after removing the effects of gender, MSI status, BRAF status, KRAS status, primary tumor location, and stage in the permutation test framework.

Biological characterization of transcriptional components associated with disease-free survival

The identified TCs associated with DFS were characterized at the individual gene and biological pathway level using several methods.

Firstly, gene set enrichment analysis (GSEA) was performed with 13 gene set collections from the Molecular Signatures Database (MsigDB) version 7.1.[6] We included all gene sets with 10 to 500 genes after filtering out genes not present in the expression profiles. Enrichment of each gene set was tested according to the two-sample Welch's t-test for unequal variance between the set of genes under investigation versus the set of genes that was not under investigation. To compare gene sets of different sizes, we transformed Welch's t statistic into a Z-score. Enrichment heatmaps were made using ClustVis. In the enrichment heatmaps, gene sets were included if the enrichment for at least one TC passed

the Bonferroni threshold for multiple testing correction. Gene sets were clustered using Spearman correlation and Ward D2.

Secondly, we used the recently developed Transcriptional Adaptation to Copy Number Alterations (TACNA) profiling method.[6] This method was used to identify TCs that capture the downstream effects of copy number alterations (CNAs) on gene expression levels.

Thirdly, we used the genetICA-network (available at https://www.genetica-network.com).[7] In short, a guilt-by-association (GBA) approach was used to predict likely functions for genes based on gene co-regulation. For this, we conducted a consensus-ICA on a large scale. A covariance matrix was calculated between 19,635 genes using the expression patterns of 135,000 gene expression profiles generated with Affymetrix HG-U133 Plus 2.0, representing the many disease states, cellular states, and genetic and chemical perturbations that were obtained. Consensus-ICA was performed on the covariance matrix. This identified a large CESs set and a mixing matrix reflecting the activity of each source in the expression pattern of the genes across the samples. Next, a GBA approach was used to predict the functionality of individual genes. First, we retrieved 16 public gene set collections describing a broad range of biological processes and phenotypes. We calculated its "bar code" by averaging the MM weight of its member genes for each gene set. Next, for each gene in the MM, the distance correlation was determined between its MM weights and the gene set bar code. A high correlation between a gene's MM weight and a gene set bar code indicated that the gene under investigation shared functionality with the genes of the specific gene set under investigation. Significance levels were obtained with permutated data (250 permutations). This strategy was applied to 23,372 well-described functional gene sets, which enabled us to create a comprehensive network of predicted functionalities of individual genes. The top and

bottom 250 genes were included if their weights were ≥ 3 or ≤ -3, respectively. Next, the enrichment of predicted functionality was calculated for the resulting gene clusters.

Determining association between transcriptional components and clinicopathological parameters

The associations between categorical variables and TCs were assessed with multinomial regression. For continuous variables, Spearman's rank correlation test was applied. All statistical analyses were performed with R version 3.6.2.

Building random survival forests

Separate random survival forests (RSFs) were built for stages 2 and 3 colon cancer using TC activities as classifiers and DFS as the response variable. For each RSF, 1,000 separate survival trees were built — each time selecting five randomly chosen TCs from the entire set of input TCs. The RSF was built with a recursive process. At each recursive step, all activity thresholds for all TCs were evaluated on their capacity to split the patients into two subgroups with divergent survival curves (assessed with a log-rank test). Next, the winner TC and its activity threshold was selected based on the most significant log-rank test. This recursive step was repeated separately for each of the two patient subgroups resulting from splitting the patients based on this winner TC and its activity threshold. A recursive step stopped if the number of total patients dropped below 50, if the number of total events (i.e., disease recurrence) was less than 25, or if splitting the patients based on the winner TC and its activity threshold resulted in a subgroup with less than 17 patients. When all recursive steps were stopped, a patient-by-patient proximity matrix was obtained for the resulting survival tree. This matrix indicates how similarly the survival tree classifies two patients.

For a RSF, all 1,000 proximity matrices are element-wise summed to get a final proximity matrix. Next, hierarchical clustering — using the Ward D2 method — was performed on this final proximity matrix to find patient subgroups of similarly classified patients based on the 1,000 survival trees in the RSF. Next, the maximum number of patient subgroups was determined for which the log-rank test showed a significant difference in survival curves.

More specifically, random survival forest analysis was performed using the following steps:

1. For each of the classifiers (C_i):
    a. If the classifier is a numeric variable,
        i. For each possible weight of the classifier as a cut-off (c):
            1. Obtain two subsets of the samples.
                a. Samples having weights < cut-off weight
                b. Or samples having weights ≥ cut-off weight
            2. Obtain survival probabilities for both of the subsets.
            3. Compare the survival probabilities using the log-rank test and obtain the log-rank statistic ($LRC_{i,c}$).
        ii. Obtain the optimum cut-off c (oc_i) for which log-rank statistic $LRC_{i,c}$ is maximum. Assign maximum of $LRC_{i,c}$ as max_LRC_i.
    b. If the classifier is a categorical variable,
        i. For each possible combination of different levels of the classifier:
            1. Obtain two subsets of samples A and B.
            2. Obtain survival probabilities for both subsets.

3. Compare the survival probabilities using the log-rank test and obtain the log-rank statistic ($LRC_{i,AB}$).

ii. Obtain the optimum combination of levels in two subsets A, B (oc_i) for which log-rank statistic $LRC_{i,AB}$ is maximum. Assign maximum of $LRC_{i,AB}$ as max_LRC_i.

2. Obtain the most significant classifier along with the optimum cut-off/combination of levels (oc) for which max_LRC_i is maximum among all classifiers.
3. Classify the samples into subsets (subset_1 and subset_2) using the most significant classifier and optimum cut-off/combination of levels.
4. In each of the subsets of the data set, repeat steps 1, 2 & 3 till the following constraints are maintained.
   a. Number of samples in subset_1 + number of samples in subset_2 ≥ 50
   b. Number of events in subset_1 + number of events in subset_2 ≥ 25
   c. Number of samples in subset_1 or number of samples in subset_2 ≥ 17

To assess the goodness-of-fit of the survival tree, the following steps were conducted:

1. A new variable called survival patient subgroups was created to store the terminal node number in the survival tree of each sample.
2. Univariate Cox proportional hazards analysis with this new variable survival patient subgroups as a predictor was performed to assess its association with DFS.
3. Concordance statistic for this Cox proportional hazards analysis was obtained to evaluate the classification power of the survival tree.

After conducting these recursive processes, obtain the number of significant classifiers (n_significant_classifiers).

1. Perform random survival forest as 1,000 survival tree analyses each time using the activity scores of n_significant_classifiers many randomly chosen TCs from all the TCs associated with disease-free survival in the univariate or multivariate disease-free survival analyses. From each tree run *r* obtain the following:
   a. List of TCs used as input in the tree ($input\_TCs_r$)
   b. List of TCs significantly appearing in the tree along with the maximum number of patients in a node ($data\_size_{TC\_r}$) where each TC significantly splits the population into two subsets.
   c. Proportion of number of patients to the total number of patients in node 1 for the maximum splitting node per significant TC ($proportion\_data\_size_{TC\_r}$).
   d. Obtain proximity matrix between all samples using the following:

   $Proximity\_matrix_r(i,j)$ is calculated as

$$\frac{\text{number of shared edges between sample i and sample j}}{\text{min (number of edges traversed by sample i, number of edges traversed by sample j)}}$$

2. From the random survival forest of 1,000 survival trees, obtain the following:
   a. Sum of all proximity matrices (Proximity_matrix_combined) showing robust proximity metric between all pairs of samples based on 1,000 survival trees.
   b. Use hierarchical clustering on the Proximity_matrix_combined matrix using Ward D2 method on spearman correlation distance to find clusters of samples performing significantly differently in terms of DFS. Use consensus clustering algorithm to find optimum number of clusters.

c. Obtain importance of each TC in the random survival forest using the following steps:

   i. Find the number of trees where the current TC was provided as an input (number_of_trees_as_input).

   ii. Find the number of trees where the current TC significantly appeared as a classifier (number_of_trees_as_classifier).

   iii. Obtain the proportion of times the current TC significantly appears in a tree when it is also provided as an input as number_of_trees_as_classifier/number_of_trees_as_input. Obtain binomial distribution based p-value for this proportion to judge if the obtained proportion is significantly different from zero.

   iv. Obtain sum of the proportions of data sizes for the current TC in all trees as $\text{proportion\_data\_size}_{TC} = \Sigma \text{proportion\_data\_size}_{TC\_r}$

   v. Obtain importance score per TC ($\text{importance\_score}_{TC}$) as:

$$\frac{\text{proportion\_data\_sizeTC}}{\text{number\_of\_trees\_as\_input}}$$

Identification of significant activity of each TC in spatial transcriptomic profiles

To identify if the activity scores of each TC in each spatial transcriptomic profile is significantly different from the null distribution of possible activity scores, we conducted the following steps:

- A set of 3,000 permutations of all the genes weights in the TC i were conducted (ith permuted TC is denoted by $TC\_permuted\_i$). For each permutation r:

a. Obtain the permuted activity score for the sample s using the following formula:

$$CMM\_permuted_r = ((TC\_permuted\_i')_{1\times p} \times TC\_permuted\_i_{p\times 1})^{-1} \times (TC\_permuted\_i')_{1\times p} \times X_{p\times n}$$

b. Thereafter a Johnson transformation was conducted on the vector $CMM\_permuted$ so that the distribution was as similar as possible to normal distribution with mean zero and standard deviation of one (referred to as $CMM\_permuted\_Johnsontransformed$). The same transformation was applied on the original CMMi,s.

c. Finally a p-value was obtained based on the position of the Johnson transformed CMMi,s with respect to the generalized normal distribution fitted to the $CMM\_permuted\_Johnsontransformed$

Log-transformed p-values were thereafter plotted in a heatmap incorporating the row and column position of the individual spatial transcriptomic profiles to visualize the location of significant activity of the TCs.

Sensitivity analysis

A sensitivity analysis was performed to determine the robustness of our results. We constructed a secondary data set for this analysis by removing all samples with available DFS data from the primary data set. We also removed samples without DFS data but which were part of a study that provided DFS data for a subset of their samples. The samples with annotated DFS removed from the primary data set were added to a third data set, referred to as the DFS data set.

To assess the robustness of the TCs, c-ICA was repeated on the secondary data set using the same parameters as used with the primary data set. TCs obtained from the primary data set were defined as primary TCs, and TCs obtained from the secondary data set were defined secondary TCs. To determine robustness, pairwise Pearson correlations were calculated between the gene weights of the primary and secondary TCs. Pairs of primary and secondary TCs with an absolute correlation coefficient above 0.5 were considered robust and referred to as robust TCs.

Next, we assessed the robustness of associations between TC activities and DFS. We performed a cross-data set projection to determine the activity of the robust secondary TCs in the DFS data set. For clarity, there was no overlap between the samples used to obtain the robust secondary TCs and the samples in the DFS data set. Therefore, the DFS data set can be considered an independent data set in this analysis. Cross-data set projection was conducted with the following steps for each cross-study transcriptional component projection analysis where transcriptional components (TCs) of a data set i (for example) were used to obtain activity scores of the same TCs in samples of another data set j (for example):

- Genes not present in both data set i and j were removed from the analysis.
- Both data set i and data set j were standardized on gene-level separately, which means each gene expression is transformed to a mean of zero and standard deviation of one.
- These standardized data sets were sample-wise merged to obtain a combined data set (Combined_i_used_for_j).
- Transcription component matrix of data set i (TC_i) and Combined_i_used_for_j were used to obtain consensus mixing matrix (CMMcombined_i_used_for_j).

$$CMMcombined\_i\_used\_for\_j=((TC\_i)'\times TC\_i)^{(-1)}\times(TC\_i)'\times Combined\_i\_used\_for\_j$$

- The subset of coefficients CMMcombined_i_used_for_j for samples from data set j is obtained as CMM_j.

Secondly, univariate and multivariate Cox proportional hazards analysis was performed with the DFS data set, as described above, to determine associations between the activity of robust secondary TC and DFS. Robust TCs significantly associated with DFS in the primary and independent DFS data sets were called robust, DFS-associated TCs.

Finally, we assessed the robustness of the RSFs. For this, the RSF building process, as described above, was performed twice, first with the DFS-associated primary TCs as input and second with the robust DFS-associated secondary TCs as input. Next, robustness was assessed by calculating the Pearson correlation between the resulting final proximity matrices.

Supplementary References

1. Clough, E. & Barrett, T. The Gene Expression Omnibus Database. *Methods Mol Biol* 1418, 93-110 (2016).
2. Becht, E. et al. Estimating the population abundance of tissue-infiltrating immune and stromal cell populations using gene expression. *Genome Biol* 17, 218 (2016).
3. Guinney, J. et al. The consensus molecular subtypes of colorectal cancer. *Nat Med* 21, 1350-1356 (2015).
4. Biton, A. et al. Independent component analysis uncovers the landscape of the bladder tumor transcriptome and reveals insights into luminal and basal subtypes. *Cell Rep* 9, 1235-1245 (2014).
5. Bhattacharya, A. et al. Transcriptional effects of copy number alterations in a large set of human cancers. *Nat Commun* 11, 715 (2020).
6. Liberzon, A. et al. The Molecular Signatures Database (MSigDB) hallmark gene set collection. *Cell Syst* 1, 417-425 (2015).
7. Urzúa-Traslaviña, C.G. et al. Improving gene function predictions using independent transcriptional components. Nat Commun 12, 1464 (2021).

Supplementary Figure 1. Random survival forest analysis of stage 2 early colon cancer patients, optimal clusters.

Shown are RSF analyses of 446 patients with stage 2 colon cancer based on the activity of 43 DFS-associated TCs as classifiers. RSF was performed as described in the Methods and Supplementary Materials. The RSF building process was performed twice, first with the DFS-associated primary TCs as the input (A), and then with the robust DFS-associated secondary TCs as the input (B). To simplify interpretation, the paired primary TCs are shown in B (indicated with an asterisk), rather than the secondary TCs; for information regarding the pairing of TCs, see Supplementary Table 2. For each RSF, an importance score was calculated for each TC, reflecting how often that TC is an important classifier in the survival trees in the forest.

DFS, disease-free survival; RFS, random survival forest; TCs, transcriptional components.

Supplementary Figure 2. Random survival forest analysis of stage 3 early colon cancer patients, optimal clusters.

Shown are RSF analyses of 290 patients with stage 3 colon cancer based on the activity of 43 DFS-associated TCs as classifiers. RSF was performed as described in the Methods and Supplementary Materials. The RSF building process was performed twice, first with the DFS-associated primary TCs as the input (A), and then with the robust DFS-associated secondary TCs as the input (B). To simplify interpretation, the paired primary TCs are shown in B (indicated with an asterisk), rather than the secondary TCs; for information regarding the pairing of TCs, see Supplementary Table 2. For each RSF, an importance score was calculated for each TC, reflecting how often that TC is an important classifier in the survival trees in the forest.

DFS, disease-free survival; RFS, random survival forest; TCs, transcriptional components.

Supplementary Figure 3. Clustering results of Random survival forest analysis of stage 2 early colon cancer patients.

Figure 3 shows the RSF analyses of 446 patients with stage 2 colon cancer based on the activity of 43 DFS-associated TCs as classifiers. Supplementary Figure 3 shows the clustering results that yielded the 10 subgroups.

DFS, disease-free survival; RFS, random survival forest; TCs, transcriptional components.

Supplementary Figure 4. Clustered activity of EMT associated DFS-associated TCs

The clustered activity of these TCs per sample is found in Supplementary Figure 4. This heatmap highlights the heterogeneity of EMT associated DFS-associated TC activity across colorectal cancer samples, signifying that distinct biological processes related to EMT are at play in different colorectal cancer samples.

DFS, disease-free survival; EMT, epithelial mesenchymal transition; TCs, transcriptional components.

Supplementary Figure 5. Spatial transcriptomic profiles in colorectal cancer samples

To pinpoint the areas of significant TC activity in spatial transcriptomic profiles, we employed a permutation-based approach. We ran 5,000 permutations for each TC-profile combination, yielding a p-value that indicates the extent to which the TC's activity in the corresponding profile differs from what would be expected by chance (the null distribution). We then transformed these p-values into logarithmic values and represented them using a heatmap. In Figure 4 the heatmap of 1 CRC sample is shown, in Supplementary Figure 5 two more heatmap of CRC samples are shown.

CRC, colorectal cancer; TCs, transcriptional components.

Supplementary Figure 6. The influence of mesenchymal cell types on EMT-related DFS-associated TCs

To get insight into the role of specific mesenchymal cells on the biology captured by these EMT-related DFS-associated TCs, we used single-cell transcriptome data obtained from the Gut Cell Atlas. We randomly selected 10% of mesenchymal Gut Cell Atlas samples for these analyses. Box and whisker plots illustrate the activity of various mesenchymal cell types in relation to the EMT-related DFS-associated TCs. The lower end of the box and the upper end of the box indicate the 1st and 3rd quartile. The vertical line that splits the box in two indicates the median. The whiskers indicate 1.5 x interquartile range below and above the 1st and 3rd quartiles respectively. Individual points indicate outliers.

DFS, disease-free survival; EMT, epithelial mesenchymal transition; TCs, transcriptional components.

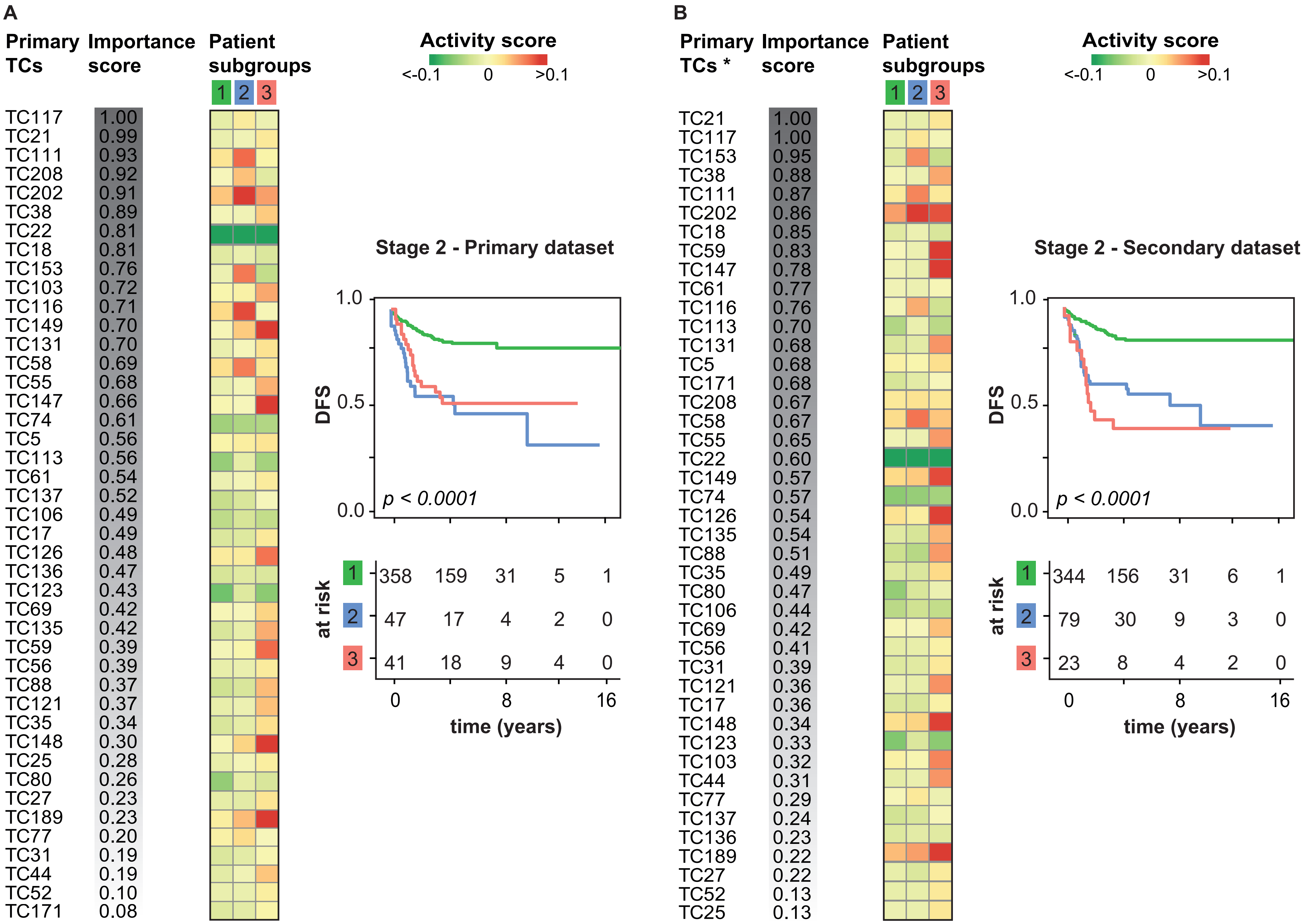
A
Primary TCs
Importance score
Patient subgroups
Activity score
<-0.1
0
>0.1
1
2
3
TC117 1.00
TC21 0.99
TC111 0.93
TC208 0.92
TC202 0.91
TC38 0.89
TC22 0.81
TC18 0.81
TC153 0.76
TC103 0.72
TC116 0.71
TC149 0.70
TC131 0.70
TC58 0.69
TC55 0.68
TC147 0.66
TC74 0.61
TC5 0.56
TC113 0.56
TC61 0.54
TC137 0.52
TC106 0.49
TC17 0.49
TC126 0.48
TC136 0.47
TC123 0.43
TC69 0.42
TC135 0.42
TC59 0.39
TC56 0.39
TC88 0.37
TC121 0.37
TC35 0.34
TC148 0.30
TC25 0.28
TC80 0.26
TC27 0.23
TC189 0.23
TC77 0.20
TC31 0.19
TC44 0.19
TC52 0.10
TC171 0.08
Stage 2 - Primary dataset
DFS
1.0
0.5
0.0
p < 0.0001
at risk
1 358 159 31 5 1
2 47 17 4 2 0
3 41 18 9 4 0
0 8 16
time (years)
B
Primary TCs *
Importance score
Patient subgroups
Activity score
<-0.1
0
>0.1
1
2
3
TC21 1.00
TC117 1.00
TC153 0.95
TC38 0.88
TC111 0.87
TC202 0.86
TC18 0.85
TC59 0.83
TC147 0.78
TC61 0.77
TC116 0.76
TC113 0.70
TC131 0.68
TC5 0.68
TC171 0.68
TC208 0.67
TC58 0.67
TC55 0.65
TC22 0.60
TC149 0.57
TC74 0.57
TC126 0.54
TC135 0.54
TC88 0.51
TC35 0.49
TC80 0.47
TC106 0.44
TC69 0.42
TC56 0.41
TC31 0.39
TC121 0.36
TC17 0.36
TC148 0.34
TC123 0.33
TC103 0.32
TC44 0.31
TC77 0.29
TC137 0.24
TC136 0.23
TC189 0.22
TC27 0.22
TC52 0.13
TC25 0.13
Stage 2 - Secondary dataset
DFS
1.0
0.5
0.0
p < 0.0001
at risk
1 344 156 31 6 1
2 79 30 9 3 0
3 23 8 4 2 0
0 8 16
time (years)

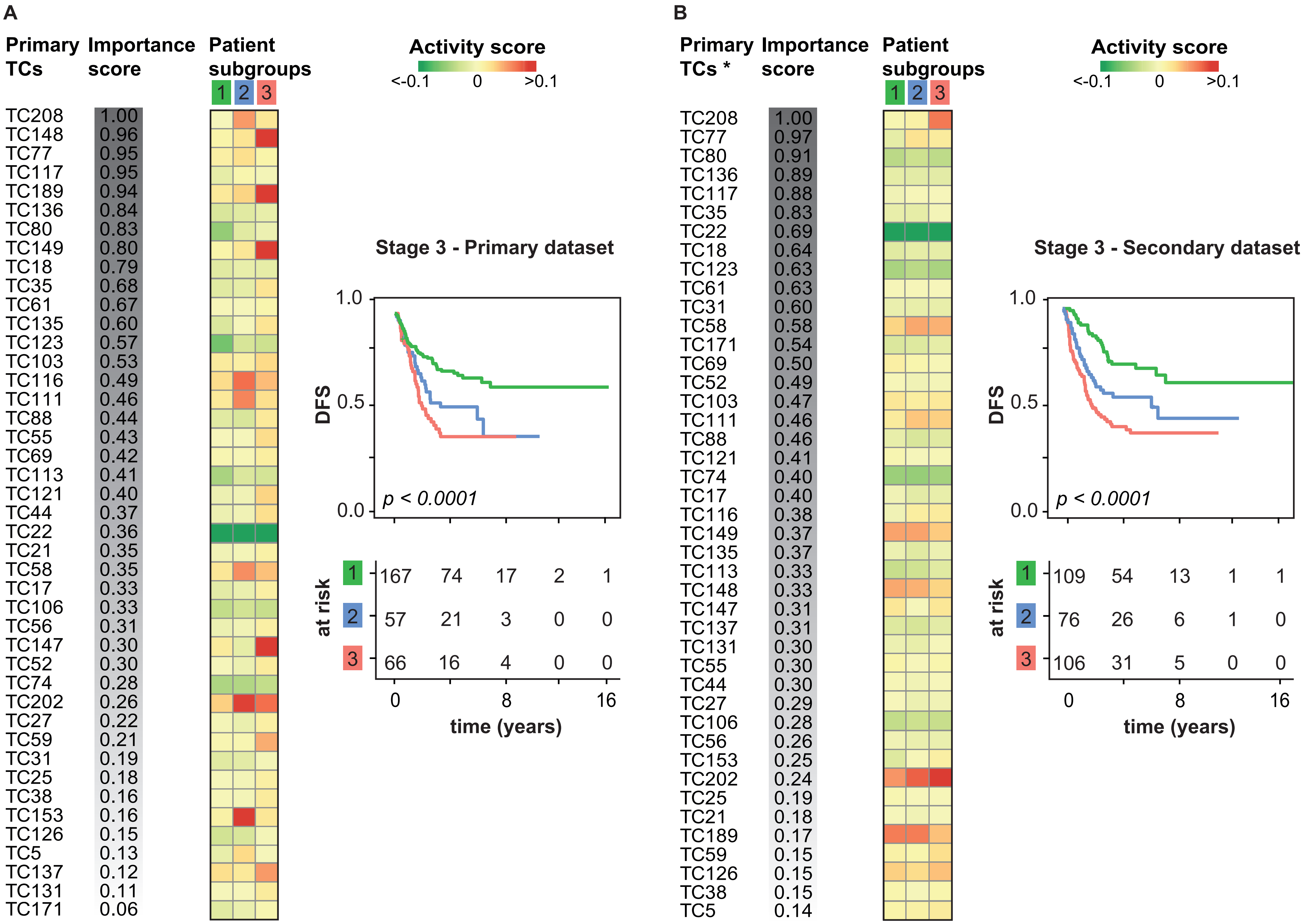
A
Primary TCs
Importance score
Patient subgroups
1 2 3
Activity score
<-0.1 0 >0.1
TC208 1.00
TC148 0.96
TC77 0.95
TC117 0.95
TC189 0.94
TC136 0.84
TC80 0.83
TC149 0.80
TC18 0.79
TC35 0.68
TC61 0.67
TC135 0.60
TC123 0.57
TC103 0.53
TC116 0.49
TC111 0.46
TC88 0.44
TC55 0.43
TC69 0.42
TC113 0.41
TC121 0.40
TC44 0.37
TC22 0.36
TC21 0.35
TC58 0.35
TC17 0.33
TC106 0.33
TC56 0.31
TC147 0.30
TC52 0.30
TC74 0.28
TC202 0.26
TC27 0.22
TC59 0.21
TC31 0.19
TC25 0.18
TC38 0.16
TC153 0.16
TC126 0.15
TC5 0.13
TC137 0.12
TC131 0.11
TC171 0.06
Stage 3 - Primary dataset
DFS
1.0
0.5
0.0
p < 0.0001
at risk
1 167 74 17 2 1
2 57 21 3 0 0
3 66 16 4 0 0
0 8 16
time (years)
B
Primary TCs *
Importance score
Patient subgroups
1 2 3
Activity score
<-0.1 0 >0.1
TC208 1.00
TC77 0.97
TC80 0.91
TC136 0.89
TC117 0.88
TC35 0.83
TC22 0.69
TC18 0.64
TC123 0.63
TC61 0.63
TC31 0.60
TC58 0.58
TC171 0.54
TC69 0.50
TC52 0.49
TC103 0.47
TC111 0.46
TC88 0.46
TC121 0.41
TC74 0.40
TC17 0.40
TC116 0.38
TC149 0.37
TC135 0.37
TC113 0.33
TC148 0.33
TC147 0.31
TC137 0.31
TC131 0.30
TC55 0.30
TC44 0.30
TC27 0.29
TC106 0.28
TC56 0.26
TC153 0.25
TC202 0.24
TC25 0.19
TC21 0.18
TC189 0.17
TC59 0.15
TC126 0.15
TC38 0.15
TC5 0.14
Stage 3 - Secondary dataset
DFS
1.0
0.5
0.0
p < 0.0001
at risk
1 109 54 13 1 1
2 76 26 6 1 0
3 106 31 5 0 0
0 8 16
time (years)

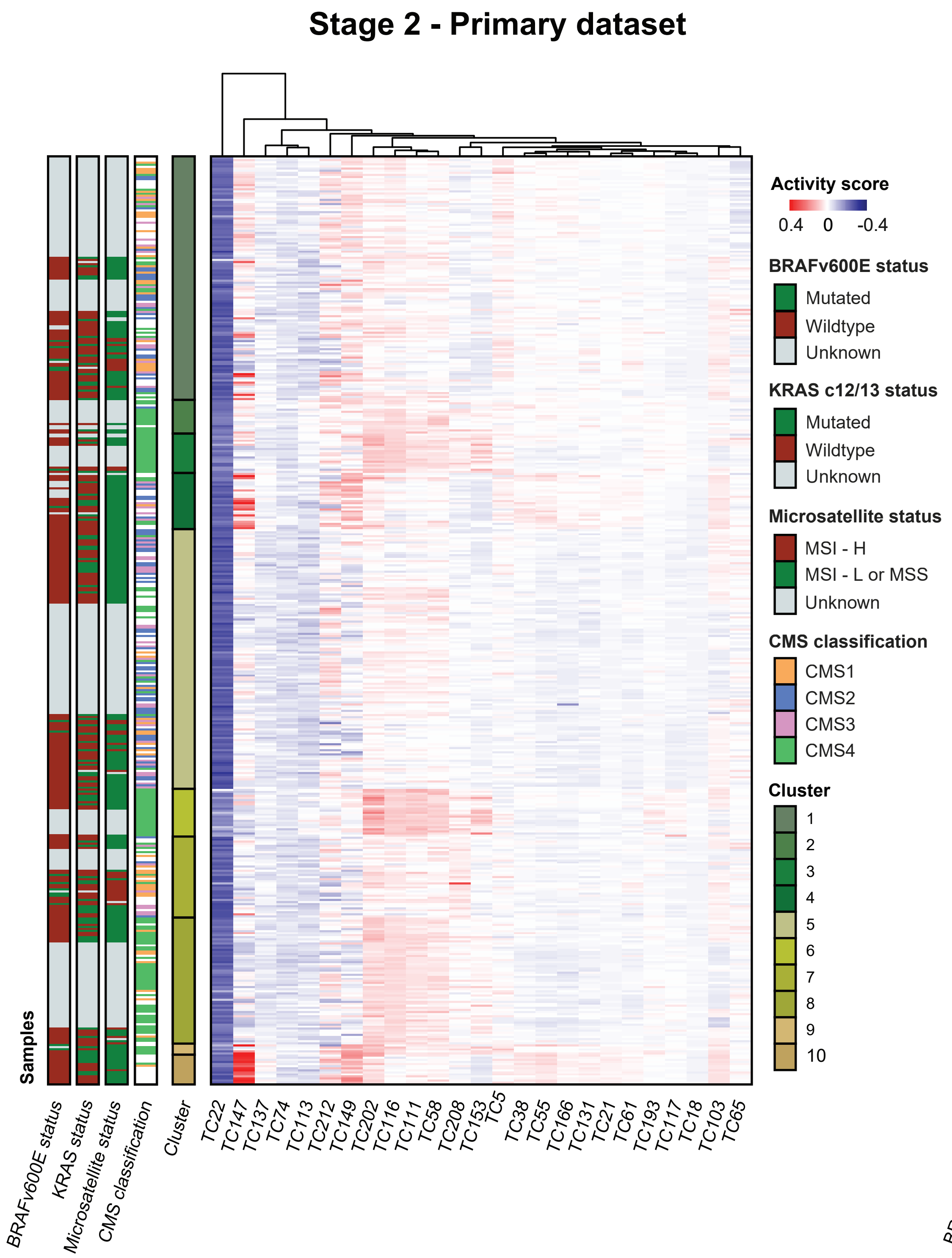

Stage 2 - Primary dataset
Activity score
0.4 0 -0.4
BRAFv600E status
Mutated
Wildtype
Unknown
KRAS c12/13 status
Mutated
Wildtype
Unknown
Microsatellite status
MSI - H
MSI - L or MSS
Unknown
CMS classification
CMS1
CMS2
CMS3
CMS4
Cluster
1
2
3
4
5
6
7
8
9
10
Samples
BRAFv600E status
KRAS status
Microsatellite status
CMS classification
Cluster
TC22 TC147 TC137 TC74 TC113 TC212 TC149 TC202 TC116 TC111 TC58 TC208 TC153 TC5 TC38 TC55 TC166 TC131 TC21 TC61 TC193 TC117 TC18 TC103 TC65

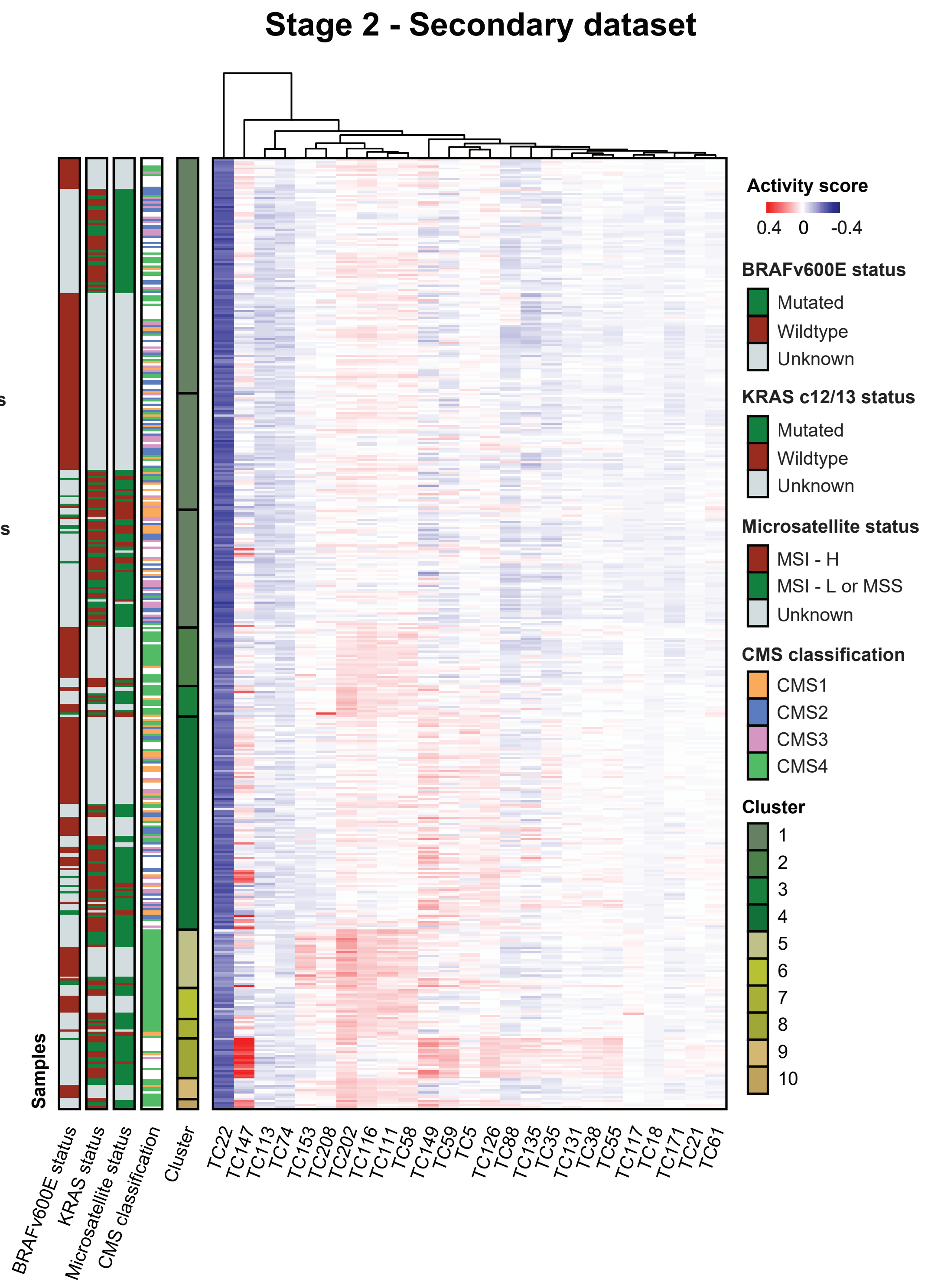

Stage 2 - Secondary dataset
Activity score
0.4 0 -0.4
BRAFv600E status
Mutated
Wildtype
Unknown
KRAS c12/13 status
Mutated
Wildtype
Unknown
Microsatellite status
MSI - H
MSI - L or MSS
Unknown
CMS classification
CMS1
CMS2
CMS3
CMS4
Cluster
1
2
3
4
5
6
7
8
9
10
Samples
BRAFv600E status
KRAS status
Microsatellite status
CMS classification
Cluster
TC22 TC147 TC113 TC74 TC153 TC208 TC202 TC116 TC111 TC58 TC149 TC59 TC5 TC126 TC88 TC135 TC35 TC131 TC38 TC55 TC117 TC18 TC171 TC21 TC61

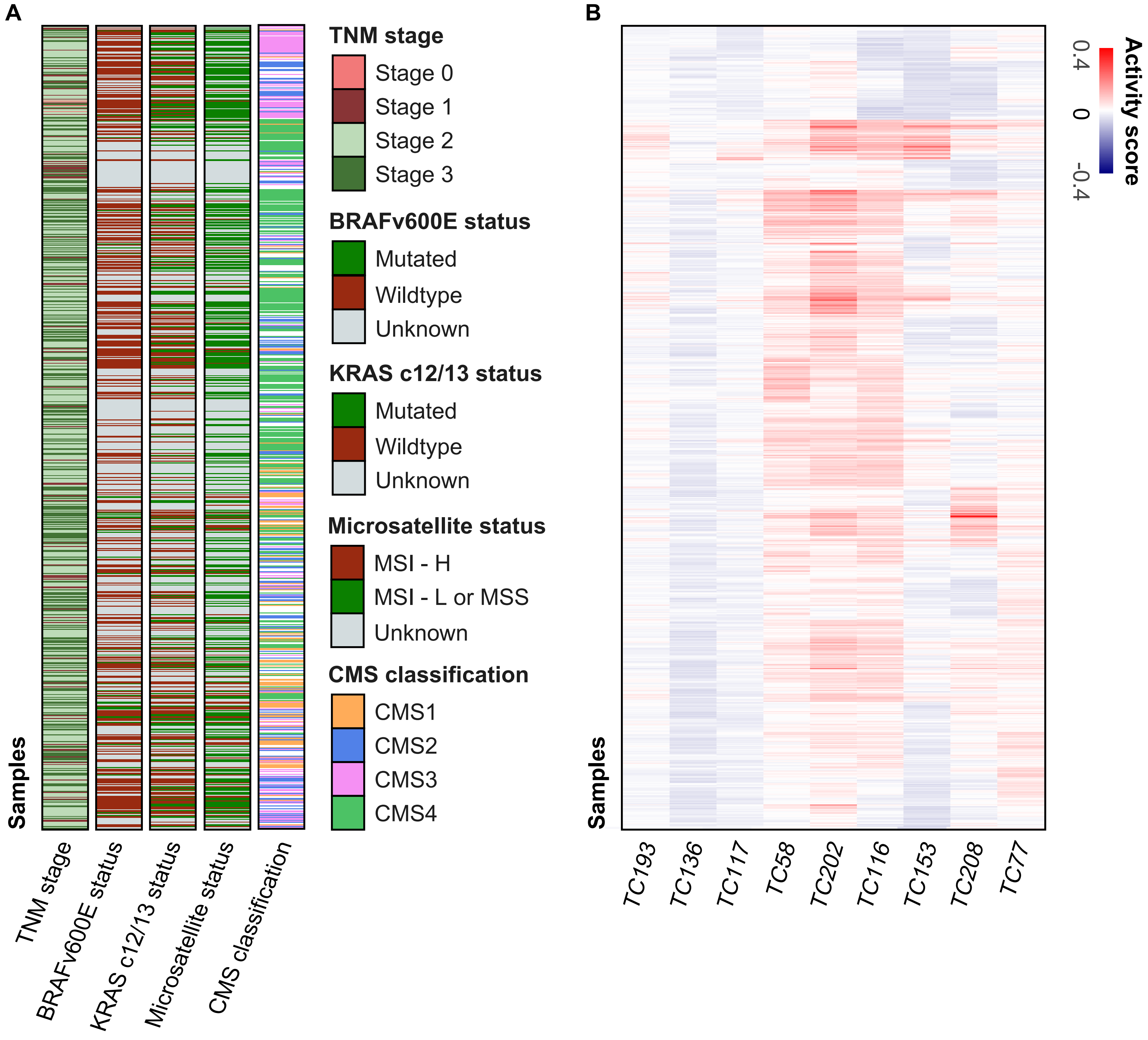

A
B
TNM stage
Stage 0
Stage 1
Stage 2
Stage 3
BRAFv600E status
Mutated
Wildtype
Unknown
KRAS c12/13 status
Mutated
Wildtype
Unknown
Microsatellite status
MSI - H
MSI - L or MSS
Unknown
CMS classification
CMS1
CMS2
CMS3
CMS4
Samples
TNM stage
BRAFv600E status
KRAS c12/13 status
Microsatellite status
CMS classification
Activity score
0.4
0
-0.4
Samples
TC193
TC136
TC117
TC58
TC202
TC116
TC153
TC208
TC77

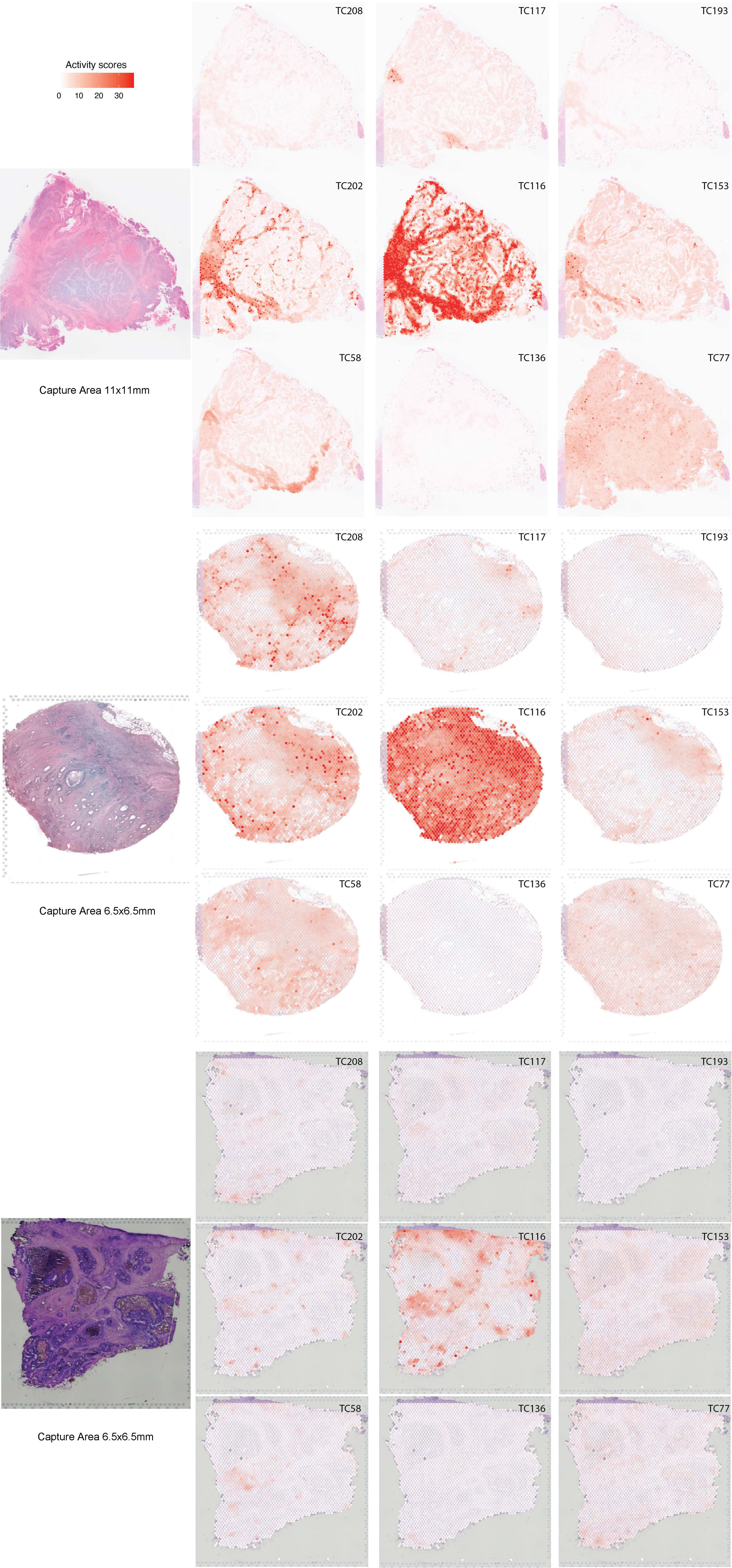

Activity scores
0
10
20
30
TC208
TC117
TC193
TC202
TC116
TC153
TC58
TC136
TC77
Capture Area 11x11mm
TC208
TC117
TC193
TC202
TC116
TC153
TC58
TC136
TC77
Capture Area 6.5x6.5mm
TC208
TC117
TC193
TC202
TC116
TC153
TC58
TC136
TC77
Capture Area 6.5x6.5mm

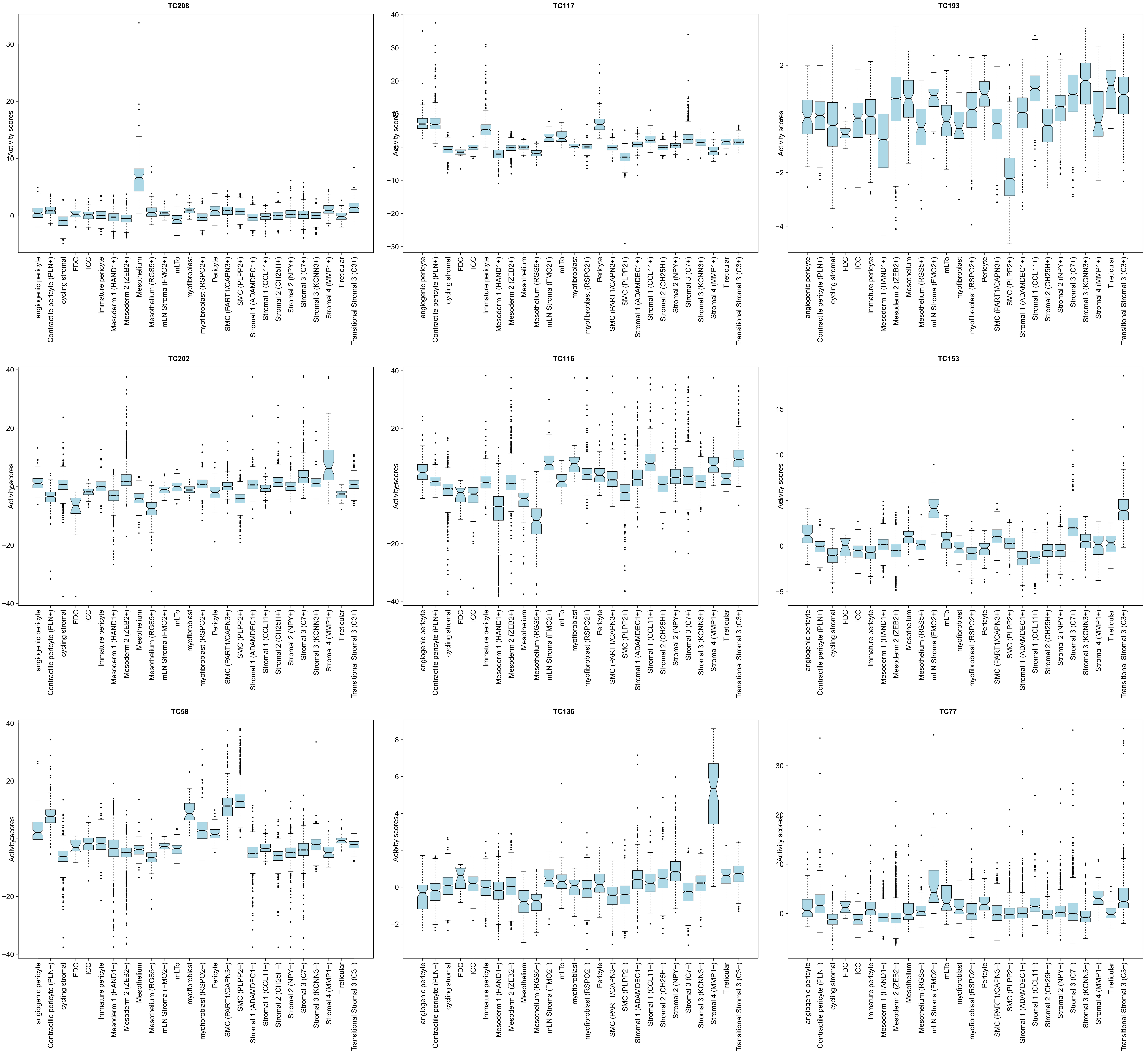
TC208
TC117
TC193
TC202
TC116
TC153
TC58
TC136
TC77
Activity scores
angiogenic pericyte
Contractile pericyte (PLN+)
cycling stromal
FDC
ICC
Immature pericyte
Mesoderm 1 (HAND1+)
Mesoderm 2 (ZEB2+)
Mesothelium
Mesothelium (RGS5+)
mLN Stroma (FMO2+)
mLTo
myofibroblast
myofibroblast (RSPO2+)
Pericyte
SMC (PART1/CAPN3+)
SMC (PLPP2+)
Stromal 1 (ADAMDEC1+)
Stromal 1 (CCL11+)
Stromal 2 (CH25H+)
Stromal 2 (NPY+)
Stromal 3 (C7+)
Stromal 3 (KCNN3+)
Stromal 4 (MMP1+)
T reticular
Transitional Stromal 3 (C3+)

Supplementary Table 1. Pairs of TCs

TC# Primary Data set	TC# Secondary Data set	Absolute correlation score
TC109	TC97	0,972644259
TC80	TC50	0,971223134
TC81	TC98	0,970003356
TC21	TC54	0,967611097
TC47	TC37	0,965393738
TC159	TC102	0,965217833
TC18	TC15	0,964799194
TC10	TC4	0,963040974
TC39	TC29	0,962584045
TC123	TC121	0,96114359
TC118	TC32	0,9609747
TC129	TC103	0,960766561
TC135	TC198	0,959555427
TC140	TC159	0,959283573
TC121	TC53	0,958930611
TC77	TC62	0,958806325
TC22	TC17	0,958102631
TC91	TC28	0,958082013
TC128	TC118	0,957323453
TC138	TC73	0,956780284
TC12	TC21	0,955742013
TC108	TC173	0,954396177
TC26	TC6	0,954089432
TC111	TC58	0,954069892
TC110	TC60	0,952281274
TC196	TC158	0,952108295
TC33	TC52	0,951824938
TC101	TC84	0,950797836
TC76	TC75	0,949947937
TC102	TC5	0,94931055
TC20	TC71	0,948554437
TC40	TC46	0,947715221
TC59	TC66	0,947690805
TC82	TC44	0,947512595
TC27	TC56	0,947088933
TC60	TC34	0,94692692
TC79	TC47	0,94670131
TC4	TC24	0,945954482
TC95	TC67	0,945898961
TC32	TC23	0,945240282
TC41	TC42	0,944755334
TC87	TC116	0,944594355
TC115	TC124	0,943372511
TC172	TC110	0,943230188
TC34	TC22	0,942375268
TC94	TC117	0,941984883
TC173	TC107	0,939479453
TC75	TC61	0,939466242
TC145	TC156	0,93818073
TC160	TC132	0,938076733
TC24	TC36	0,935851188
TC53	TC31	0,93557428
TC83	TC78	0,935482625
TC45	TC90	0,934915344
TC25	TC30	0,93471767
TC54	TC10	0,933912353
TC106	TC120	0,933662515
TC17	TC19	0,933550755
TC7	TC38	0,932752131
TC5	TC35	0,932730539
TC88	TC129	0,932684108
TC99	TC149	0,93241623
TC103	TC119	0,932052076
TC9	TC8	0,931355169
TC29	TC11	0,93130402
TC70	TC106	0,930967916
TC113	TC155	0,930605046
TC176	TC196	0,930367405
TC98	TC133	0,930279986
TC57	TC18	0,929929503
TC150	TC111	0,929844942
TC52	TC1	0,929714256
TC1	TC7	0,929429372
TC144	TC105	0,928832149
TC55	TC13	0,928757368
TC124	TC88	0,927898671
TC46	TC41	0,92762644
TC42	TC104	0,925825175
TC143	TC134	0,924923937
TC156	TC143	0,924907515
TC189	TC128	0,924647045
TC30	TC113	0,924630248
TC96	TC49	0,924227098
TC163	TC109	0,923758211
TC107	TC148	0,923512008
TC68	TC91	0,923172115
TC35	TC40	0,922829287
TC31	TC63	0,92254271
TC180	TC92	0,922028447
TC61	TC65	0,920806915
TC125	TC86	0,920501315
TC48	TC70	0,92048056
TC49	TC59	0,920010588
TC69	TC3	0,91996245
TC202	TC157	0,919911712
TC97	TC87	0,919600274
TC122	TC188	0,919558986
TC133	TC96	0,917979508
TC65	TC57	0,917961221
TC44	TC48	0,917195356
TC2	TC33	0,917050838
TC127	TC215	0,916935778
TC36	TC100	0,916488497
TC16	TC25	0,916454086
TC58	TC72	0,915015344
TC14	TC114	0,914856876
TC139	TC164	0,91331495
TC19	TC26	0,913177476
TC186	TC140	0,913067711
TC149	TC160	0,913052761
TC66	TC79	0,912606825
TC142	TC137	0,912294091
TC28	TC20	0,912205125
TC11	TC101	0,911825597
TC117	TC99	0,911327239
TC130	TC187	0,910686841
TC155	TC127	0,910676732
TC74	TC74	0,91055956
TC15	TC27	0,909879608
TC13	TC14	0,909843762
TC218	TC161	0,908962033
TC84	TC85	0,907933835
TC63	TC9	0,907657843
TC179	TC178	0,907036855
TC165	TC180	0,906470808
TC171	TC176	0,904526121
TC182	TC181	0,902984834
TC198	TC171	0,902113073
TC195	TC184	0,901947451
TC50	TC69	0,901933931
TC38	TC55	0,901580348
TC90	TC145	0,901256612
TC178	TC183	0,900437096
TC185	TC192	0,900356667
TC64	TC43	0,899110946
TC100	TC81	0,898671926
TC67	TC82	0,898090347
TC72	TC122	0,898062443
TC78	TC64	0,897734451
TC6	TC2	0,896626164
TC161	TC191	0,895680751
TC85	TC94	0,893684489
TC43	TC209	0,893588437
TC104	TC80	0,892866035
TC169	TC153	0,892736035
TC147	TC169	0,890854142
TC137	TC83	0,889781806
TC8	TC45	0,889461722
TC73	TC76	0,889035211
TC23	TC12	0,888335258
TC105	TC123	0,888135494
TC148	TC162	0,887458969
TC119	TC93	0,886609395
TC183	TC182	0,886004034
TC164	TC179	0,885649335
TC126	TC200	0,884597275
TC56	TC126	0,88451894
TC213	TC206	0,884086161
TC3	TC51	0,882547895
TC177	TC195	0,880489312
TC194	TC207	0,880407312
TC215	TC217	0,878398103
TC116	TC152	0,874914915
TC112	TC77	0,8748116
TC89	TC68	0,873278958
TC168	TC146	0,871777953
TC132	TC142	0,870826388
TC92	TC39	0,868503483
TC157	TC224	0,866492549
TC114	TC163	0,866347476
TC37	TC89	0,866315953
TC152	TC216	0,863595777
TC71	TC125	0,863003272
TC93	TC151	0,858793178
TC131	TC112	0,852661985
TC134	TC177	0,850288165
TC51	TC16	0,849950718
TC151	TC205	0,848851972
TC204	TC218	0,848506865
TC153	TC115	0,848154102
TC136	TC141	0,847134277
TC170	TC172	0,84635837
TC154	TC201	0,843234049
TC191	TC210	0,840205835
TC212	TC231	0,840035633
TC181	TC212	0,828979636
TC62	TC131	0,828357471
TC141	TC147	0,821963527
TC199	TC228	0,801209881
TC146	TC135	0,733438937
TC208	TC185	0,70488951

Supplementary Table 2. Important scores TCs

Stage 2 Primary		Stage 3 Primary		Stage 2 Secondary			Stage 3 Secondary		
TC# Primary	Importance score	TC# Primary	Importance score	TC# Secondary	TC# Paired primary	Importance score	TC# Secondary	TC# Paired primary	Importance score
TC117	1	TC208	1	TC54	TC21	1	TC185	TC208	1
TC21	0,994649409	TC148	0,956333938	TC99	TC117	0,997359708	TC62	TC77	0,9696
TC111	0,934442519	TC77	0,950118092	TC115	TC153	0,953801981	TC50	TC80	0,909449602
TC208	0,916485159	TC117	0,949957948	TC55	TC38	0,878699552	TC141	TC136	0,890748528
TC202	0,912478738	TC189	0,941925572	TC58	TC111	0,871502837	TC99	TC117	0,879761521
TC38	0,888141505	TC125	0,887088123	TC157	TC202	0,862387892	TC40	TC35	0,833034483
TC193	0,855775058	TC136	0,839023558	TC15	TC18	0,845376089	TC17	TC22	0,693479003
TC166	0,8187106	TC80	0,825453253	TC66	TC59	0,826008969	TC15	TC18	0,642875732
TC22	0,813410874	TC149	0,797662186	TC169	TC147	0,776419325	TC121	TC123	0,634561727
TC18	0,805602804	TC18	0,786642885	TC65	TC61	0,76638565	TC65	TC61	0,627641379
TC212	0,805032387	TC65	0,73362069	TC152	TC116	0,75635975	TC63	TC31	0,603732996
TC153	0,759631064	TC193	0,707921715	TC155	TC113	0,697955461	TC72	TC58	0,576692209
TC103	0,717786218	TC35	0,680846705	TC112	TC131	0,680291112	TC176	TC171	0,543894856
TC116	0,709467893	TC61	0,67338241	TC35	TC5	0,679102309	TC3	TC69	0,499582027
TC149	0,69882952	TC135	0,595827586	TC176	TC171	0,678545174	TC1	TC52	0,494585129
TC131	0,697780269	TC123	0,573509507	TC185	TC208	0,670123318	TC119	TC103	0,474778325
TC58	0,694887892	TC216	0,56387931	TC72	TC58	0,667787743	TC58	TC111	0,463472688
TC55	0,68042016	TC103	0,525932442	TC13	TC55	0,649721303	TC129	TC88	0,457320024
TC147	0,655734186	TC116	0,488908638	TC17	TC22	0,603449807	TC53	TC121	0,410403772
TC65	0,615470852	TC111	0,463832288	TC160	TC149	0,570689594	TC74	TC74	0,404350133
TC74	0,605381166	TC88	0,439978448	TC74	TC74	0,570282856	TC19	TC17	0,400756939
TC5	0,560214923	TC55	0,42886057	TC200	TC126	0,543384739	TC152	TC116	0,377924589
TC113	0,559541604	TC69	0,416978654	TC198	TC135	0,542455572	TC160	TC149	0,368422482
TC61	0,540711443	TC113	0,408505747	TC129	TC88	0,50906695	TC198	TC135	0,366028097
TC137	0,518772064	TC121	0,401461769	TC40	TC35	0,492152466	TC155	TC113	0,326680304
TC106	0,491297784	TC44	0,36994622	TC50	TC80	0,467057606	TC162	TC148	0,325542784
TC17	0,488892325	TC22	0,356968391	TC120	TC106	0,436507937	TC169	TC147	0,313044893
TC126	0,483183857	TC21	0,351018809	TC3	TC69	0,417210219	TC83	TC137	0,312053148
TC136	0,467855081	TC58	0,350689655	TC126	TC56	0,413464512	TC112	TC131	0,304324477
TC123	0,434998533	TC220	0,348607427	TC63	TC31	0,385135969	TC13	TC55	0,303834998
TC69	0,417232543	TC212	0,345517241	TC53	TC121	0,360909892	TC48	TC44	0,295240809
TC135	0,415044843	TC17	0,334522394	TC19	TC17	0,358890226	TC56	TC27	0,288073394
TC220	0,411413418	TC106	0,333014681	TC162	TC148	0,341699884	TC120	TC106	0,2772578
TC216	0,398570628	TC56	0,30679803	TC121	TC123	0,330332386	TC126	TC56	0,263049941
TC59	0,393017297	TC147	0,300623624	TC119	TC103	0,317125774	TC115	TC153	0,246067973
TC56	0,392632928	TC52	0,30058117	TC48	TC44	0,307712263	TC157	TC202	0,240421456
TC88	0,373365097	TC74	0,276342209	TC62	TC77	0,29361435	TC30	TC25	0,189591901
TC121	0,37280659	TC202	0,257471264	TC83	TC137	0,235919694	TC54	TC21	0,175507573
TC35	0,341650757	TC115	0,239877091	TC141	TC136	0,232564075	TC128	TC189	0,165967016
TC115	0,334835501	TC27	0,224655172	TC128	TC189	0,219945408	TC66	TC59	0,154326019
TC148	0,301439698	TC59	0,205383533	TC56	TC27	0,215678611	TC200	TC126	0,153097841
TC25	0,278379604	TC31	0,186386494	TC1	TC52	0,129641956	TC55	TC38	0,15015674
TC80	0,260551986	TC25	0,183494769	TC30	TC25	0,12872835	TC35	TC5	0,140996169
TC27	0,229618834	TC180	0,17966954						
TC189	0,227500777	TC157	0,178562526						
TC77	0,201732293	TC38	0,160957854						
TC31	0,1901392	TC153	0,159678683						
TC44	0,185934093	TC126	0,153914259						
TC157	0,141876925	TC166	0,150274863						
TC125	0,133432985	TC5	0,134638086						
TC180	0,125350336	TC137	0,123807777						
TC52	0,100141079	TC131	0,105241379						
TC171	0,078428133	TC171	0,055281307						
