# Supplementary material for: Independent transcriptional patterns reveal biological processes associated with disease-free survival in early colorectal cancer
Source: Commun Med (Lond). 2024 May 3;4:79. doi: 10.1038/s43856-024-00504-z (PMC11068726; doi:10.1038/s43856-024-00504-z)
Supplement: Supplementary file 3 — Description of Additional Supplementary Files [file 43856_2024_504_MOESM3_ESM.pdf]

Description of Additional Supplementary Files

File name: Supplementary Data 1

File Description: 1,995 early CRC 13 1 samples and all 1,368 non-malignant samples for a sensitivity analysis.

File name: Supplementary Data 2

File Description: Source data for the figures.
