# Supplementary material for: Independent transcriptional patterns reveal biological processes associated with disease-free survival in early colorectal cancer
Source: Commun Med (Lond). 2024 May 3;4:79. doi: 10.1038/s43856-024-00504-z (PMC11068726; doi:10.1038/s43856-024-00504-z)
Supplement: Supplementary file 6 — Reporting Summary [file 43856_2024_504_MOESM6_ESM.pdf]

Corresponding author(s): Rudolf Fehrmann

Last updated by author(s): Apr 9, 2024

Reporting Summary

Nature Portfolio wishes to improve the reproducibility of the work that we publish. This form provides structure for consistency and transparency in reporting. For further information on Nature Portfolio policies, see our Editorial Policies and the Editorial Policy Checklist.

Statistics

For all statistical analyses, confirm that the following items are present in the figure legend, table legend, main text, or Methods section.

n/a	Confirmed	
☐	☒	The exact sample size (*n*) for each experimental group/condition, given as a discrete number and unit of measurement
☐	☒	A statement on whether measurements were taken from distinct samples or whether the same sample was measured repeatedly
☐	☒	The statistical test(s) used AND whether they are one- or two-sided<br>*Only common tests should be described solely by name; describe more complex techniques in the Methods section.*
☐	☒	A description of all covariates tested
☐	☒	A description of any assumptions or corrections, such as tests of normality and adjustment for multiple comparisons
☐	☒	A full description of the statistical parameters including central tendency (e.g. means) or other basic estimates (e.g. regression coefficient) AND variation (e.g. standard deviation) or associated estimates of uncertainty (e.g. confidence intervals)
☐	☒	For null hypothesis testing, the test statistic (e.g. *F*, *t*, *r*) with confidence intervals, effect sizes, degrees of freedom and *P* value noted<br>*Give P values as exact values whenever suitable.*
☒	☐	For Bayesian analysis, information on the choice of priors and Markov chain Monte Carlo settings
☐	☒	For hierarchical and complex designs, identification of the appropriate level for tests and full reporting of outcomes
☐	☒	Estimates of effect sizes (e.g. Cohen's *d*, Pearson's *r*), indicating how they were calculated

Our web collection on statistics for biologists contains articles on many of the points above.

Software and code

Policy information about availability of computer code

Data collection	No software was used
Data analysis	R version 3.5.2

For manuscripts utilizing custom algorithms or software that are central to the research but not yet described in published literature, software must be made available to editors and reviewers. We strongly encourage code deposition in a community repository (e.g. GitHub). See the Nature Portfolio guidelines for submitting code & software for further information.

Data

Policy information about availability of data

All manuscripts must include a data availability statement. This statement should provide the following information, where applicable:

- Accession codes, unique identifiers, or web links for publicly available datasets
- A description of any restrictions on data availability
- For clinical datasets or third party data, please ensure that the statement adheres to our policy

Microarray expression data was collected from the public data repository: Gene Expression Omnibus with accession number GPL570 (generated with Affymetrix HG-U133 Plus 2.0). Single-cell data in h5ad format were obtained from the Gut Cell Atlas (https://www.gutcellatlas.org/#datasets). Three publicly available colorectal cancer spatial transcriptomic profiles were used obtained from the 10x website (https://www.10xgenomics.com). Specifically, https://www.10xgenomics.com/datasets/human-colorectal-cancer-11-mm-capture-area-ffpe-2-standard, https://www.10xgenomics.com/datasets/human-intestine-

cancer-1-standard, and https://www.10xgenomics.com/datasets/human-colorectal-cancer-whole-transcriptome-analysis-1-standard-1-2-0. The composition of the primary transcriptional components and the gene set enrichment analysis results are publicly available and browsable by gene and gene set at our online portal available at http://transcriptional-landscape-colon.opendatainscience.net. Source data for the figures are available as Supplementary Data 2. For any other data inquiries, please contact the corresponding author.

The complete set of codes utilized in this study is available at https://github.com/arkajyotibhattacharya/TranscriptionalLandscapeColorectalCancer.

Human research participants

Policy information about studies involving human research participants and Sex and Gender in Research.

Reporting on sex and gender	For all selected samples, publicly available corresponding clinicopathological data were collected from GEO or the corresponding manuscript, including gender. Apart from reporting patient characteristics, this data was not used for any analyses.
Population characteristics	For all selected samples, publicly available corresponding clinicopathological data were collected from GEO or the corresponding manuscript. This data included disease-free survival data, stage, primary tumor location, age, gender, microsatellite instability status, received adjuvant treatment or not, tumor protein P53 status, Kirsten rat sarcoma virus status, and v-Raf murine sarcoma viral oncogene homolog B status.
Recruitment	Not applicable
Ethics oversight	Not applicable

Note that full information on the approval of the study protocol must also be provided in the manuscript.

Field-specific reporting

Please select the one below that is the best fit for your research. If you are not sure, read the appropriate sections before making your selection.

☒ Life sciences ☐ Behavioural & social sciences ☐ Ecological, evolutionary & environmental sciences

For a reference copy of the document with all sections, see nature.com/documents/nr-reporting-summary-flat.pdf

Life sciences study design

All studies must disclose on these points even when the disclosure is negative.

Sample size	All relevant available publicly available raw micro array expression profiles were obtained form the Gene Expression Omnibus. Data acquisition was restricted tot he Affymetrix HG-U133 Plus 2.0 platform. No formal sample-size calculation was performed. The sample size was sufficient to realize the aim of the study as we were able to associate identified transcription components with disease-free survival.
Data exclusions	Any duplicate samples were identified and excluded from out analysis
Replication	A sensitivity analysis was performed to determine the robustness of our results. In order to do so, we created a secondary dataset by excluding all samples from the primary dataset that had available disease-free survival data. The samples from the primary dataswet that had annotated disease-free survival were then added to a separate dataset, which we refer to as the disease-free survival dataset.
Randomization	Not applicable
Blinding	Not applicable

Reporting for specific materials, systems and methods

We require information from authors about some types of materials, experimental systems and methods used in many studies. Here, indicate whether each material, system or method listed is relevant to your study. If you are not sure if a list item applies to your research, read the appropriate section before selecting a response.

Materials & experimental systems

n/a	Involved in the study
☒	☐ Antibodies
☒	☐ Eukaryotic cell lines
☒	☐ Palaeontology and archaeology
☒	☐ Animals and other organisms
☒	☐ Clinical data
☒	☐ Dual use research of concern

Methods

n/a	Involved in the study
☒	☐ ChIP-seq
☒	☐ Flow cytometry
☒	☐ MRI-based neuroimaging
